# Highly Transparent Structural Colors with Iridescent Sheen via Burst-Mode Laser Processing

**DOI:** 10.3390/mi17060648

**Published:** 2026-05-25

**Authors:** Quanxin Yang, Minghui Fan, Siyu Xue, Kezhao Ma, Sha Li, Jiao Geng, Liping Shi

**Affiliations:** 1 Hangzhou Institute of Technology, Xidian University, Hangzhou 311200, China; 2 School of Optoelectronic Engineering, Xidian University, Xi’an 710071, China

**Keywords:** femtosecond laser processing, structure color, surface protrusion structure

## Abstract

The growing demand for structural coloration methods that simultaneously exhibit an iridescent sheen effect and a base color on transparent substrates calls for a single-step fabrication procedure capable of periodic and localized modulation of thin-film structure. In this work, a composite thin-film structure consisting of aluminum nitride-aluminum (AlN-Al)-soda-lime glass substrate is designed, deposited, and subsequently processed using burst-mode femtosecond laser. By systematically varying the number of sub-pulses, the pulse-to-pulse distance, and the average laser power while maintaining a fixed single-sub-pulse energy (1 μJ), the precise control over thermal accumulation and surface protrusion morphology is achieved, resulting in a series of highly transparent structural colors with iridescent sheen effects. Reflectance spectra, transmittance data, confocal microscopy, scanning electron microscopy and coupled energy dispersive spectrometer analyses, and the finite-difference time-domain simulations reveal that the observed color variation originates from laser-induced air gaps between the Al and AlN layers, rather than from compositional changes, and that the resulting periodic surface protrusion structures govern the iridescent sheen effect. The proposed method enables large-scale patterning while preserving high transmittance, as demonstrated by the desired hue, saturation, and iridescent sheen. This burst-mode laser processing strategy offers a material- and production line-compatible route for realizing coupled interference- and diffraction-based structural colors, with promising applications in decorative purposes with anti-counterfeiting or encryption purposes, where both angle-independent base color and angle-dependent iridescent sheen effect are required.

## 1. Introduction

Coloration of transparent materials [1] not only holds decorative value but also possesses numerous application values, including anti-counterfeiting [2], encryption [3], and surface thermal radiation modulation [4], and has long served as a platform for novel coloration methods. Structural color, emerging from these coloration methods, offers advantages such as non-toxicity, environmental friendliness, resistance to fading, and ease of artificial tunability due to its physical coloration mechanism [5,6,7,8]. At present, structural coloration methods are primarily based on the following three physical mechanisms: interference, diffraction, and scattering [9]. Additionally, owing to these different mechanisms, the applicable fields of these structural colors also vary as follows: structural colors based on interference rely on thin-film structures and are suitable for decorative purposes or surface thermal radiation modulation [10,11]; those based on diffraction rely on grating structures and are suitable for information security fields like anti-counterfeiting and encryption [12,13,14]; and those based on scattering rely on metal or dielectric particle structures and are also suitable for decorative purposes.

However, current industrial development poses new demands for structural color methods involving complex and coupled mechanisms, such as exhibiting an iridescent sheen effect while simultaneously presenting a base color [15,16,17], which imposes limitations on the processing procedures. Essentially, a processing method that can achieve periodic and localized modulation of thin-film structure in a single fabrication procedure is needed. In this context, laser-induced formation of surface protrusion structure becomes a feasible option [18,19,20,21]. In this paper, a composite thin-film structure based on aluminum nitride-aluminum (AlN-Al), and glass substrate is designed, deposited, laser-processed, and then characterized. The resulting structural colors are highly correlated with the surface protrusion structures formed by single laser pulses, i.e., with laser power and thermal accumulation. The required iridescent sheen effect can be modulated by the pulse-to-pulse distance. To modulate the thermal accumulation behavior while maintaining a constant laser peak power, the burst-mode sub-pulse train is utilized. In this way, various structural colors with high transmittances and accompanying iridescent sheen effects can be achieved.

## 2. Materials and Methods

### 2.1. Materials

**Figure 1:**
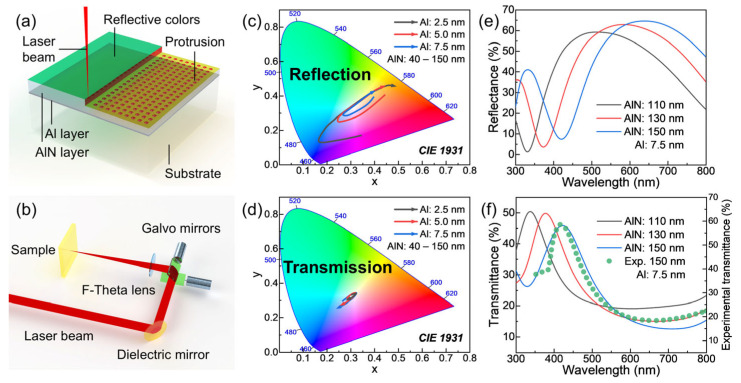
The schematic diagrams of (**a**) the AlN-Al-glass substrate composite thin-film structure with the laser-induced periodic surface protrusion structures and (**b**) the femtosecond laser processing. The simulated (**c**) reflection and (**d**) transmission CIE chromatic diagrams of this composite thin-film structure with different layer thicknesses (Al layer thickness: 2.5, 5, 7.5 nm; AlN layer thickness: 40–150 nm). The arrows indicate the direction of increasing AlN layer thickness. Several representative (**e**) reflectance and (**f**) transmittance spectra. Symbols correspond to the experimental transmittance spectrum.

To verify the material and production line compatibilities of this coloration method, mature and widely used soda-lime glass (70 mm × 70 mm × 0.7 mm, AS2, Asahi Glass Co., Ltd., Tokyo, Japan) is selected as the substrate material. Before the subsequent magnetron sputtering procedure, the glass substrates are ultrasonically cleaned in aqueous ethanol solution (~1:1, *v*/*v*) for 15 min to remove potential surface contaminants. Subsequently, a high-vacuum magnetron sputtering system (PVD500, SKY Technology Development Co., Ltd., Shenyang, China) is utilized to sequentially deposit the Al and AlN layers, with commercially purchased sputtering targets (Al: 99.999%, AlN: 99.9%, Beijing Zhongnuo Advanced Material Technology Co., Ltd., Beijing, China), DC power of 100 W, and Ar flow of 30 sccm. As a result, the AlN-Al-glass substrate composite thin-film structure can be fabricated, as illustrated in Figure 1a. Both material preparation and film deposition procedures are conducted at room temperature.

### 2.2. Burst-Mode Laser Processing

The as-deposited composite thin-film structure is subsequently treated by the burst-mode femtosecond laser beam generated by a fiber amplification solution laser (OR-50-HE, Ultron Photonics Technology Co., Ltd., Hangzhou, China). The output laser beam, with a wavelength of 1030 nm and a diameter of 3 mm, is first expanded and collimated by a 6× beam expander and then focused onto the composite thin-film structure surface by an f-theta scan lens (f = 163 mm) coupled with galvo mirrors (Neutron20D, Anshan precision optical scanning technology Co., Ltd., Anshan, China). The schematic diagram of the femtosecond laser processing is shown in Figure 1b.

Unless otherwise specified, typical laser processing parameters used are listed in Table 1. Additionally, to ensure that the periods of the structures formed after laser processing in two directions are consistent, the following three parameters need to be coupled: line spacing, pulse-to-pulse distance, and scanning speed. In brief, the pulse-to-pulse distance in the horizontal direction should match the line spacing in the vertical direction. This can be realized by setting the scanning speed to the product of the pulse-to-pulse distance and the laser repetition rate. It should be noticed that only the concepts of intra-pulse and sub-pulse, when explicitly mentioned, are used to describe the burst-mode; all other parameters (like pulse-to-pulse distance) are used to describe the single giant pulse that constitutes the overall burst-mode sub-pulse train.

### 2.3. Characterization

Based on an optical metallurgical microscope (RX50M, Sunny Optical Technology (group) Co., Ltd., Ningbo, China), the optical microscopy photographs, the reflectance and transmittance spectra are captured by a high-resolution digital camera and a microscope-coupled spectrometer (SR-6VIS400-25, Ocean Optics Inc., Orlando, FL, USA), respectively. The following three different objective lenses are replaced to accommodate different requirements: a low-magnification objective (LMPlanFL, 5×, N.A. = 0.15) is used for capturing the overall colors, an objective (LMPlanFL, 20×, N.A. = 0.40) is used for detailed observation and spectrum measurement, while a high-magnification objective (LMPlanFL, 100×, N.A. = 0.80) is also used for detailed observation. Unless otherwise specified, the measured spectra are all normalized using pristine glass samples as the reference.

Further surface morphology characterization of the laser-treated composite thin-film structure is conducted by a confocal laser scanning microscope (LSM 900, Carl Zeiss AG, Oberkochen, Germany) with a specialized apochromatic objective (Epiplan-Apochromat, 100×, N.A. = 0.95), while the elemental composition analyses are performed by using a field emission scanning electron microscopy (SEM, GeminiSEM 450, Carl Zeiss AG, Oberkochen, Germany) and the integrated energy-dispersive spectrometer (EDS, Ultim Extreme, Oxford Instruments Plc, High Wycombe, UK).

### 2.4. Optical Simulation

The following two different simulation methods are used: the finite-difference time-domain (FDTD) method and the finite element method (FEM). Both methods are performed based on commercial software (Ansys Lumerical FDTD 2024R1 and COMSOL Multiphysics 6.3). The FDTD method is utilized to design appropriate film thicknesses by evaluating parameters like color gamut, saturation, and transmittance. The raw data obtained directly from the simulations are reflectance and transmittance spectra, while the quantitative color metrics can be calculated from the corresponding spectrum data. Used optical parameters of the designed thin films are obtained from measurements of the actual deposited films by a high-precision ellipsometer (SE-VM-L, Wuhan Eoptics Technology Co., Ltd., Wuhan, China). To simplify the calculation process, the boundary conditions in the x- and y-directions are set as anti-symmetric and symmetric, respectively. Also, to avoid possible oscillations and convergence difficulties, perfectly matched layers are configured in the z-direction boundaries, and the z-direction meshes at the metallic Al layer are refined to 0.05 nm with 10 nm buffers on both sides.

Meanwhile, the FEM is utilized to investigate the thermal diffusion and accumulation behaviors during the interaction of femtosecond laser pulses with the composite thin-film structure. Furthermore, the effects of the sub-pulses within a burst-mode pulse train are also incorporated. Considering the differences in the absorption coefficients of femtosecond laser pulses by different thin-film layers, the absorption process of laser pulse energy can be idealized as a heat source confined within the metallic Al layer electron subsystem by the two-temperature model. Additionally, the event interface is utilized to simplify the computational process while maintaining relatively high accuracy.

## 3. Results and Discussion

### 3.1. Composite Thin-Film Structure Design

To simultaneously meet the requirements for high transmittance and structural color, the well-established thin-film interference structural coloration method is adopted. This composite thin-film structure, from top to bottom, consists of: a transparent interference layer, which provides the interference phase difference and protects the underlying structure; a metallic reflective layer, which enhances the overall reflectance of the structure to improve the brightness and saturation of the structural color and also provides a reflective phase shift to reduce the required thickness of the transparent interference layer; and a transparent substrate material. The additional requirement for iridescent sheen effect can be realized by the laser-induced periodic surface protrusion structures. The schematic diagram of the overall structure is shown in Figure 1a.

The periodic, stable, and regular fabrication of this specific surface protrusion structure relies on a localized laser-matter interaction process. This imposes constraints on both selected materials of the designed composite thin-film structure and the type of laser used as follows: the thermal conductivities of the constituent thin-film materials must be sufficiently high to rapidly dissipate residual heat; meanwhile, the pulse duration of the laser must be short enough to avoid continuous heating of the composite thin-film structure. Based on this rule, the pulsed laser with a pulse duration of 750 fs and the AlN–Al–glass substrate composite thin-film structure are selected. Compared with the well-established titanium–titanium dioxide (Ti-TiO_2_) system, or with replacing AlN with aluminum oxide (Al_2_O_3_), the AlN–Al system still offers significant advantages in thermal conduction efficiency.

Considering the coloration mechanism of thin-film interference structural color, the thickness of the metallic reflective layer determines the brightness and saturation of the structural color, while the specific hue is determined by the thickness of the transparent interference layer. For the quantitative analysis of the structural color and the subsequent selection of specific film thickness parameters, the reflection and transmission spectra of this composite thin-film structure with different layer thicknesses (Al layer thickness: 2.5, 5, 7.5 nm; AlN layer thickness: 40–150 nm) are simulated. Corresponding CIE chromatic diagrams are calculated and illustrated in Figure 1c,d, and the arrows indicate the direction of increasing AlN layer thickness. Figure 1e,f exhibit several representative reflectance and transmittance spectra, respectively. For these composite thin-film structures with such layer thicknesses, the overall transmission colors are significantly weaker than the reflection ones, which satisfies the requirement for transmission transparency. In the reflection case, smaller Al layer thickness corresponds to higher saturation of the achievable structural color. However, it is worth mentioning that the high peak temperature during femtosecond laser processing can lead to the oxidation of the Al layer by the glass substrate; therefore, the deposited Al layer thickness needs to be larger than that expected for the final structural colors. Based on the pre-experiment results, thicknesses of 7.5 nm for Al and 150 nm for AlN are considered appropriate. Corresponding transmittance spectrum of the actually deposited composite thin-film structure is also shown in Figure 1f, which is in good agreement with the simulation result.

### 3.2. Structural Colors

The as-deposited composite thin-film structures are subsequently used in the burst-mode laser processing procedure. Here, two different parameter scanning strategies are adopted, and unless otherwise specified, the laser processing parameters are consistent with those in Section 2.2.

**Figure 2:**
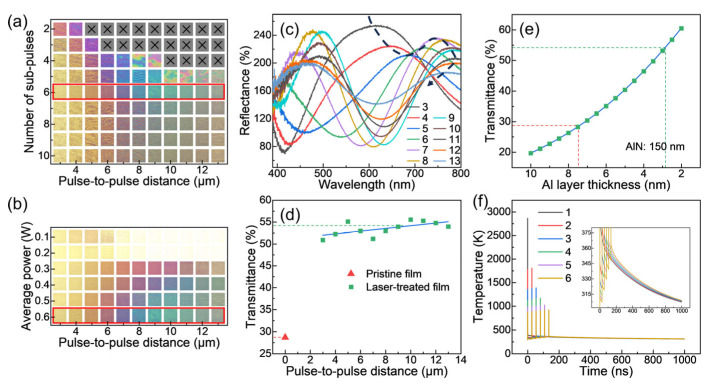
The color patches fabricated (**a**) under fixed average laser power (0.6 W) and different numbers of sub-pulses and pulse-to-pulse distances; (**b**) under single sub-pulse energy (1 μJ) and different average powers and pulse-to-pulse distances. The color patches presented here are essentially microscopic images at a scale of 1 mm × 1 mm. The (**c**) reflectance spectra and (**d**) transmittance data of the color patches marked by red boxes in (**a**,**b**). (**e**) The simulated transmittance data of the composite thin-film structure as a function of the Al layer thickness, with the AlN layer thickness fixed at 150 nm. (**f**) The temperature evolution of the composite thin-film structure during the laser processing under the strategy with a fixed average laser power.

First, under a fixed average laser power (0.6 W), increasing the number of sub-pulses can cause the energy of the original single pulse to be distributed among more sub-pulses, thereby significantly reducing the energy per sub-pulse and corresponding peak power. The variation in the peak power of the laser sub-pulses profoundly affects the peak temperatures attainable in different layers of the composite thin-film structure, thus directly determining whether thermal ablation and the induction of the surface protrusion structure occur. The corresponding fabricated color patches are shown in Figure 2a, and the color patches presented here are essentially microscopic images at a scale of 1 mm × 1 mm. It can be observed that under this laser processing condition, six sub-pulses are most suitable, with the corresponding single sub-pulse energy being 1 μJ. When the single sub-pulse energy exceeds this specific value, thermal ablation occurs, causing partial delamination of the film surface. Meanwhile, when the single sub-pulse energy is below this specific value, the peak temperatures attainable in different layers of the composite thin-film structure are insufficient, resulting in irregularly formed surface protrusion structures and low saturations of the structural colors.

Subsequently, this single sub-pulse energy value is fixed (1 μJ) to ensure that the induced surface protrusion structure meets the requirements while avoiding thermal ablation. Then, by adjusting the number of sub-pulses within the burst-mode pulse train and the spatial spacing of the single giant pulse constituted by the burst-mode pulse train (so-defined pulse-to-pulse distance), the thermal accumulation can be precisely controlled, thereby generating a series of different structural colors, as exhibited in Figure 2b. Also, the color patches presented here are essentially microscopic images at a scale of 1 mm × 1 mm. The color patches fabricated under 0.6 W average laser power with single sub-pulse energy 1 μJ are selected for further analyses, as marked by the red boxes in Figure 2a,b. Corresponding reflectance spectra and transmittance data are illustrated in Figure 2c and Figure 2d, respectively. Since this structural color is intrinsically introduced by laser-induced surface protrusion structures, the color is distributed radially along the structures. As a result, the macroscopically observed color arises from the superposition of these microscopic colors. Consequently, the relationship between the final superimposed color and the morphology of the laser-induced surface protrusion structures, thermal accumulation, and the pulse-to-pulse distance is rather complex and difficult to describe quantitatively.

As inferred above, during the femtosecond laser processing, the Al layer indeed suffers oxidation by the glass substrate, resulting in a decrease in its thickness, which manifests as an increase in the overall transmittance of the composite thin-film structure. After oxidation, the residual Al thickness can also be evaluated from the transmittance of the overall structure. Figure 2e shows the simulated transmittance data of the composite thin-film structure as a function of the Al layer thickness, with the AlN layer thickness fixed at 150 nm. It can be seen that after laser processing, the Al layer thickness decreases from ~7.5 nm to ~2.8 nm, which indicates that the aforementioned design of the Al layer thickness meets the requirements. For such thin-film interference structural colors, a transmittance greater than 50% is generally defined as a high-transmittance condition, in order to balance the saturation and brightness of the realized colors against the overall structural transmittance. Therefore, the fabricated structural color patches meet the transmittance requirement.

**Figure 3:**
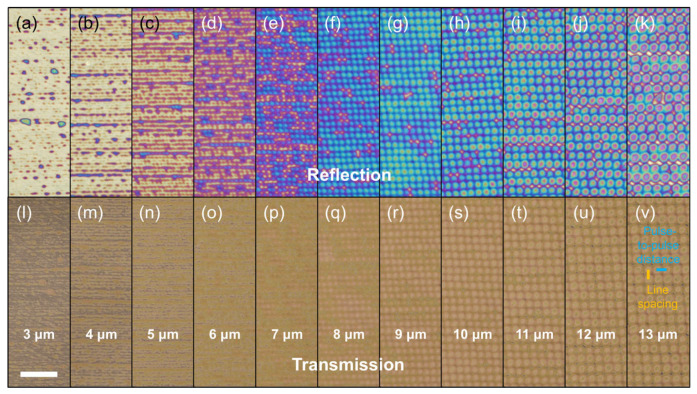
The (**a**–**k**) reflection and (**l**–**v**) transmission microscopy images of the macroscopic color patches marked by red boxes in Figure 2a,b fabricated under different pulse-to-pulse distances. The schematic diagram in (**v**) presents the pulse-to-pulse distance in the horizontal direction and the line spacing in the vertical direction. The scale bar in (**l**) is 50 μm and all sub-figures share the same scale bar.

The reflection and transmission microscopy images corresponding to these macroscopic color patches are shown in Figure 3. It can be observed that after burst-mode laser processing, laser-induced periodic surface protrusion structures are formed on the surface of this composite thin-film structure, with a period consistent with the adopted pulse-to-pulse distance. Under transmission conditions, the overall colors of this composite thin-film structure do not vary significantly with the laser processing parameters, meeting the requirement for transmission transparency. The schematic diagram of the pulse-to-pulse distance in the horizontal direction and the line spacing in the vertical direction is presented in Figure 3v.

To further understand the effect of the number of sub-pulses within the burst-mode pulse train, the temperature evolution of the composite thin-film structure during the laser processing under the strategy with a fixed average laser power is simulated, and the results are exhibited in Figure 2f. It can be seen that under this strategy, increasing the number of sub-pulses can significantly reduce the peak temperature and prolong the time that the overall structure spends in the intermediate temperature region. This can be understood as effectively extending the time required for thermal diffusion, thereby precisely enhancing the thermal accumulation behavior.

### 3.3. Surface Protrusion Structure

To further analyze this formed surface protrusion structure, color patches are refabricated under slightly reduced single sub-pulse energy (pulse-to-pulse distance: 13 μm, number of sub-pulses: six, average laser power: 0.5 W, and corresponding single pulse energy: 0.83 μJ) so that these structures can become separated from each other.

**Figure 4:**
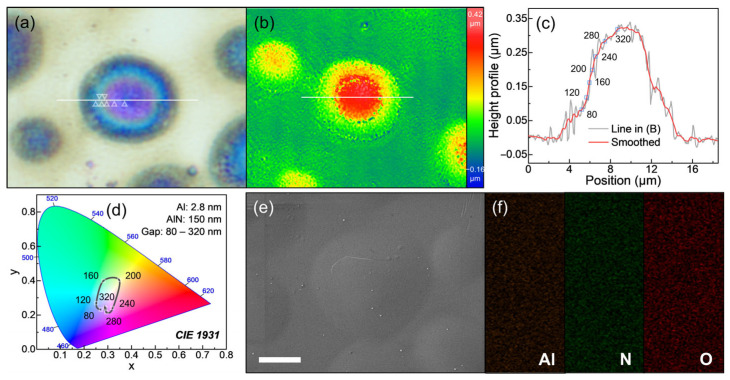
The (**a**) reflection microscopy image, (**b**) two-dimensional surface morphology distribution, and (**c**) corresponding one-dimensional height profile marked by white line in (**b**) of the surface protrusion structures. The squares in (**c**) indicate the positions with specific height increments while the triangles in (**a**) indicate the colors with specific positions. (**d**) The simulated reflection CIE chromatic diagram of this composite thin-film structure with fixed Al and AlN layer thicknesses (Al: 2.8 nm, AlN: 150 nm) and different air gap thicknesses (80–320 nm). The arrow indicates the direction of increasing air gap thickness while the squares indicate the colors with specific air gap thicknesses. The (**e**) SEM and (**f**) combined EDS results of the surface protrusion structures. The scale bar in (**e**) is 5 μm, and (**a**,**b**,**e**,**f**) share the same scale bar.

As shown in Figure 4, several surface protrusion structures are selected for a series of characterizations, and the reflection microscopy image is exhibited in Figure 4a. It can be observed that this surface protrusion structure presents a series of different structural colors radially from the center to the periphery, while maintaining the same color in the angular direction. The two-dimensional surface morphology distribution and one-dimensional height profile of the surface protrusion structure, measured by confocal microscopy, are shown in Figure 4b,c, respectively. It can be seen that this color distribution should correspond to the variation in surface height. To verify whether this color distribution is caused by the surface height variation in the composite thin-film structure or by possible laser-induced oxidation of the AlN layer, SEM and combined EDS measurements are performed, and the results are illustrated in Figure 4e,f. It can be observed that in and around the surface protrusion structure, the element distributions of Al, nitrogen (N), and oxygen (O) present no significant change, indicating that the composition of the AlN layer does not change significantly, thereby ruling out the possibility of oxidation. Therefore, the color variation in this surface protrusion structure is entirely caused by its surface height variation.

Given the differences in thermal diffusion and thermal expansion coefficients among the different layers of the composite thin-film structure, this surface height difference should be ascribed to the difference in the thickness of the air gap introduced between the Al layer and the AlN layer during the laser processing. To verify this inference, the reflection spectra corresponding to different air gap thicknesses are simulated with fixed Al and AlN layer thicknesses (Al: 2.8 nm, AlN: 150 nm), and the corresponding CIE chromatic diagrams are calculated and shown in Figure 4d. Several specific air gap thickness values are selected, and the corresponding positions on the surface protrusion structure are derived from the height profile in Figure 4c and marked in Figure 4a. The actual structural colors and the simulated ones are listed in Table 2, which present good agreement.

**Figure 5:**
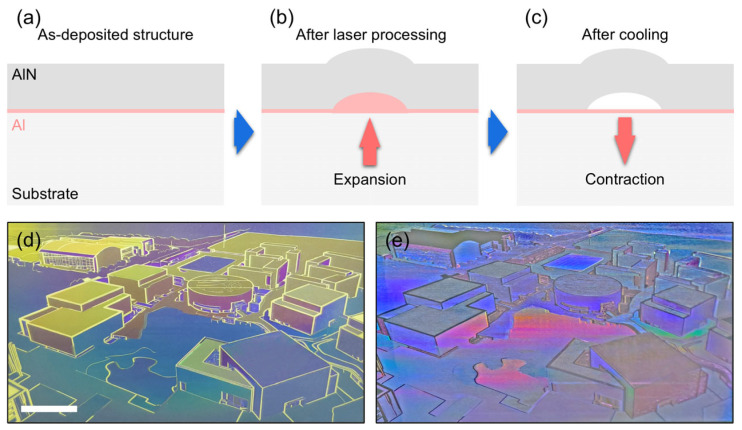
The phenomenological model of the thermal expansion behavior: (**a**) as-deposited structure, (**b**) after laser processing, and (**c**) after cooling. The large-scale patterning sample images of (**d**) reflection and (**e**) iridescent examples. The scale bar in (**d**) is 10 mm, and (**d**,**e**) share the same scale bar.

To clearly express the process of this thermal expansion behavior, a phenomenological model, as exhibited in Figure 5a–c is established as follows: when the femtosecond laser pulse is initially absorbed by the Al layer, the Al layer undergoes a dramatic temperature increase. Since its thermal expansion coefficient is much higher than that of the AlN layer and the glass substrate, the Al layer presents strong expansion, exerting compressive stress on the AlN layer and causing it to suffer plastic deformation. As a result, the thermal expansion of the underlying Al layer leads to a dome-like protrusion on the composite thin-film structure. Meanwhile, due to the high thermal conductivity of both the Al and AlN layers, the heat in the Al layer is rapidly diffused and subsequently dissipated. After its temperature quickly decreases, the Al layer undergoes contraction. At this moment, since the AlN layer has undergone plastic deformation, it cannot recover its intimate contact with the Al layer, leaving an air gap between the Al layer and the AlN layer.

Experimentally, this structural coloration method is stable and repeatable; the resulting colors are consistent with the laser processing parameters used. To demonstrate the large-scale patterning capability of this structural coloration method, an example pattern is designed and fabricated using the aforementioned laser processing parameters, and the results are shown in Figure 5d,e. During the capture of the reflection example in Figure 5d, the sample is placed on flocked fabric to minimize unexpected reflected light. As for the iridescent example in Figure 5e, the sample is suspended, with the light source positioned above the sample. The hue, saturation, and iridescent sheen effect of the patterned sample all meet the design specifications.

## 4. Conclusions

In summary, a burst-mode femtosecond laser processing strategy for generating highly transparent structural colors with iridescent sheen effects on an AlN-Al-glass substrate composite thin-film structure is demonstrated. This composite thin-film structure is deposited by magnetron sputtering, and systematic experiments are conducted to investigate the effects of the number of sub-pulses, pulse-to-pulse distance, and average laser power on the resulting coloration. By fixing the single sub-pulse energy at an optimal value (1 μJ) to avoid thermal ablation while ensuring the formation of periodic surface protrusion structures, precise control over thermal accumulation is achieved, yielding a series of distinct structural colors with high transmittance. Reflectance spectra, transmittance data, confocal microscopy, SEM/EDS analyses, and thermal FEM simulations reveal that the color variation originates from laser-induced air gaps between the Al and AlN layers, rather than from compositional changes. The proposed method enables large-scale patterning, which exhibits the desired hue, saturation, and iridescent sheen. This work provides a single-step, material-compatible route to simultaneously realize thin-film interference colors and diffraction-based iridescent sheen effects. The approach holds promises for applications in decorative coatings with anti-counterfeiting or encryption purposes, where both base color and angle-dependent iridescent sheen effect are required.

## Figures and Tables

**Table 1:** Typical laser processing parameters.

Fixed Parameters									
Laser pulse duration		Repetition rate			Focal length			Focal spot diameter	
750 fs		100 kHz			163 mm			11.9 μm	
**Variable Parameters**									
Average laser power	Single pulse energy			Line spacing		Pulse-to-pulse distance			Scanning speed
0.1–0.6 W	1–6 μJ			3–13 μm		3–13 μm			300–1300 mm/s
**Intra-Burst Parameters**									
Number of sub-pulses			Intra-burst repetition rate				Intra-burst pulse separation		
1–10			37 MHz				27 ns		

**Table 2:** The comparison of experimental and simulated colors.

Gap (nm)	80	120	160	200	240	280	320
Measured Color	Blue	Blue	Green	Yellow	Purple	Purple	Blue
Simulated Color	Blue-purple	Blue	Green	Yellow	Purple	Purple	Purple-blue

## Data Availability

Data underlying the results presented in this paper can be obtained from the corresponding author upon reasonable request.
